# Ethnopharmacology of Love

**DOI:** 10.3389/fphar.2018.00567

**Published:** 2018-07-03

**Authors:** Marco Leonti, Laura Casu

**Affiliations:** ^1^Department of Biomedical Sciences, University of Cagliari, Cagliari, Italy; ^2^Department of Environmental and Life Sciences, University of Cagliari, Cagliari, Italy

**Keywords:** lovesickness, infatuation, sex, ethnomedicine, serotonin, dopamine, aphrodisiacs, beta-caryophyllene

## Abstract

**Background:** Elixirs conferring eternal youth or inducing amatory and erotic attraction have been searched for without success. Lovesickness is a widespread affliction resulting from unrequited love and/or the impossibility for physical and emotional union. The symptoms are reflections of altered dopamine, serotonin, noradrenaline, testosterone and cortisol levels and range from frenzy and intrusive thinking to despair and depression, sharing traits with the neurochemistry of addiction and compulsive behavior disorder. Although it can seriously impact the quality of life, lovesickness is currently not considered in official disease classification systems. Consequently, no official therapeutic guidelines exist, leaving subjects to seek the cure on their own.

**Methods:** We review literature of the past 2000 years dealing with the concept, diagnosis and the healing of lovesickness and contextualize it with neurochemical, ethnomedical, and ethnographic data. Since neurobiological and pharmacological connections between the love drive and the sex drive exist, we review also the literature about herbal an- and aphrodisiacs, focusing on their excitatory or calmative potential.

**Results:** An overall consensus regarding socio-behavioral regimes exists for dealing with lovesickness from historical through contemporary literature. The herbal drugs used for treating lovesickness or inducing love passion do not possess the alleged properties. The pharmacological effects of aphrodisiacs are heterogeneous, including dopaminergic and adrenergic activities, but there is no evidence for any serotonergic effects. The libido-regulating properties of anaphrodisiacs seem to be associated with sedative and toxic effects or decreasing testosterone levels. CB_2_ receptors expressed on dopaminergic neurons of the ventral tegmental area, part of the brain’s reward circuit, implicated with addiction, orgasm and strong emotions such as love, might constitute a new therapeutic target.

**Conclusion:** The common food additive and CB_2_ agonist β-caryophyllene might have the potential to attenuate dopaminergic firing, quenching the reward and thus motivation associated with romantic love. From Greek mythology to modern history, cultural expressions and implications of love, sex and procreation is and was organized along hierarchical lines that put men on top. The neuronal predispositions and activities associated with falling in love will probably forever remain nature’s and Eros’ secret.

**GRAPHICAL ABSTRACT:**
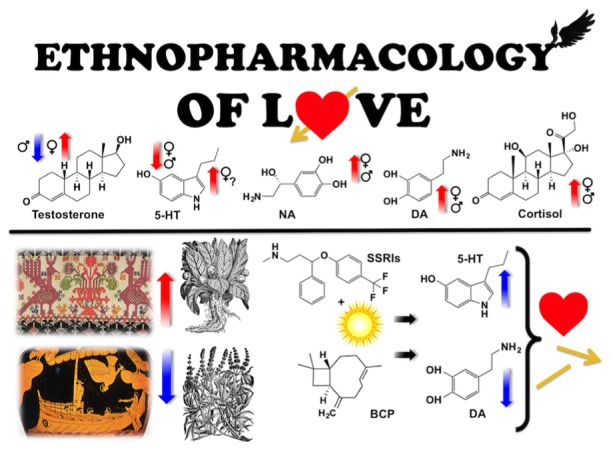
A cure for infatuation?

“So whoever you are who call for help from my art, put no faith in witchcraft and incantations”.

(Ovid – *Remedia Amoris*)

## Introduction

Being struck by Eros’ golden arrow penetrating one’s heart symbolizes the overwhelming emotions we are inescapably exposed to when falling in love. Eros (‘*Cupido*’ or ‘*Amor*’ in ancient Rome) is a playful god distributing love darts apparently without following any rules. Eros was associated not only with romantic feelings but also with sexual attraction. In Greek mythology the deity was able to excite love in and between men, women, semi-human figures, animals, planets and even rivers and fountains with the consequence that a woman could fall in love with a river or a fountain with a boy (Cantarella, 2009, p. 15). The perceived intentionality and causality synthesized through the symbolism of the love arrow (Págan Cánovas, 2011) and experienced through the intoxicating and aching effect of erotic infatuation has led to the belief that mutual feeling of passionate love can be triggered with love potions, herbal drugs, charms and sorcery (e.g., Lewin, 1920; Taberner, 1985; Cabot and Cowan, 1992; Faraone, 1999; Saar, 2017).

The concept of arrows of love linking extreme passion and illness might have been derived from the arrows of the glance (“inescapable glance” from the ancient Greek tragedy ‘Prometheus Bound’ ca. 430 BC; Págan Cánovas, 2011). The idea that love passion or lovesickness would enter the human body through the eyes was also expressed by Hesiod (around 700 BC), Plato (ca. 425-347 BC) and Aristoteles (ca. 383-322 BC; Ferrand, 1990, p. 232–234). This theory coincides with the notion that the eyes are generally seen as the windows to the soul (Ibn Hazm, 1994; Lick et al., 2016) and the fact that they reflect our emotions via the innervation of the sympathetic system through the interaction of noradrenaline with α1-receptors (McCorry, 2007). Strong feelings such as pleasure and fear, as well as pleasurable visual stimuli are processed by the sympathetic-adrenal system and are generally reflected by pupillary dilatation (Hess and Polt, 1960). Specifically, sexual arousal is associated with pupil dilation, which can serve as a visual cue to other subjects in assessing their own sexual appeal (Lick et al., 2016). It is said that dilated pupils were pharmacologically induced for signaling seductive cues or sexual arousal in the Italian Renaissance period^1^. According to what seems to be an urban legend (see footnote 1), Venetian women used the sap of *Atropa belladonna* L. (Solanaceae) for dilating their pupils to produce the ‘look of love’ effect, as the anticholinergic L-hyoscyamine (**2A**) contained in the juice mimics the mydriatic action of noradrenalin (**2B**) physiologically released during sexual arousal. Conversely, beauty lies in the eye of the beholder and according to Andreas Capellanus (1150–1220 AD), blindness impedes love (Capellanus, 2006, p. 11).

Although they share common brain activation patterns, sexual arousal and the experience of romantic love are distinct from each other (Cacioppo et al., 2012) because their corresponding drives evolved in different contexts (Diamond, 2004). The sex drive is a consequence of sexual mating strategy, whereas romantic love evolved out of the child–parent (“infant–caregiver”) relationship (Diamond, 2004). The ‘bonding hormone’ oxytocin is in fact released in women and men during orgasm as well as in women when giving birth and during lactation (Carter and Porges, 2013; Veening et al., 2015). From an evolutionary perspective, romantic love is an efficiency-raising motivation system that directs one’s attention and mating energy on one specific potential partner (Fisher et al., 2005).

In any event, the simple scheme of an arrow launched by the bow of a love god that intoxicates the psyche and hurts the body stands in gross contrast to the complexity of human emotions and their fundamental neurochemistry and psychology (Esch and Stefano, 2005; Zeki, 2007; Young, 2009; Págan Cánovas, 2011). The feeling of romantic love (also ‘infatuated love’ or ‘limerence’; see Tennov, 1998) is the strongest sensation known to humankind and is characterized by a mix of unbearable exhilarating joy, anxiety, obsessive thinking and craving for emotional and physical union (Fromm, 1973; Tennov, 1998; Fisher, 2004; Stendhal, 2014). Romantic love is very likely recognized and acknowledged by all human cultures (Jankowiak and Fischer, 1992; Hatfield et al., 2007; Oghia, 2015) although the level of experienced intensity varies greatly between individuals (Tennov, 1998), probably reflecting personal life history as well as genetic and cultural factors.

The arbitrariness by which Eros distributes his love darts, however, implies that reciprocity is by no means guaranteed. Unrequited love, erotic frustration and the craving for the beloved object manifest themselves in what is commonly referred to as lovesickness (see Tennov, 1998). This often depressive and melancholic state of mind is characterized by intrusive thinking and also has an addictive component. Lovesickness has been pathologized in previous centuries but is currently not included in the ICD-10 (International Classification of Diseases; World Health Organization [WHO], 2018a), the ICPC (International Classification of Primary Care; World Health Organization [WHO], 2018b) or the DSM-5 (Diagnostic and Statistical Manual of Mental Disorders; American Psychiatric Association, 2018) and thus represents a discarded diagnosis (Bynum, 2001). Love addiction is to be distinguished from drug addiction. Although behavioral, anatomical and neurochemical parallels exist, such as the association with elevated dopamine (**2C**) levels (Abbott, 2002; Burkett and Young, 2012), love addiction is reversible and can be re-experienced serially with different love objects over the course of life (Reynaud et al., 2010). Despite lovesickness no longer being perceived as a state of disease, associated despair, depression and jealousy are known to trigger a range of harmful behaviors such as stalking, self-injury, physical abuse, homicide and suicide (Tennov, 1998; Fisher, 2004; Toohey, 2004, pp. 59–103; Earp et al., 2013; Marazziti et al., 2015).

Generally in literature and specifically in folklore and mythology, far more recommendations and recipes focus on how to kindle romantic feelings and mutual attraction in the love object than to end passion and overcome lovesickness (Tennov, 1998). The most frequently reported cure for lovesickness from classical antiquity until the Renaissance period, and proposed by a range of authors such as Ovid (43 BC – 17/18 AD; “I also urge you to have two girls at once”), Galen (129–199 AD), Oribasius (326–403), Paul of Aegina (ca. 626–690), Avicenna (ca. 980–1037), Rhazes (ca. 865–925), Constantinus Africanus (ca. 1020–1087), Hildegard of Bingen (1098–1179), Gerard of Cremona (1114–1187), and others, was sexual intercourse (Ferrand, 1990, pp. 123–128; Kline, 2001, p. 251; Allen, 2002; McNamara, 2016). In fact, Galen classified lovesickness as a sort of melancholia (depression) caused by excess of black bile and associated body liquids such as blood and semen (Mesulam and Perry, 1972; Ferrand, 1990; Allen, 2002). Therefore, the evacuation of the excess liquids through bloodletting and sex was considered an appropriate cure (Allen, 2002).

The first treatise on fighting lovesickness and unrequited attraction known in history is ‘*Remedia Amoris*’ by the Roman poet Ovid (43 BC-17/18 AD). Although Ovid advised against the use of herbal remedies and magic: – “If anyone thinks he can be helped by harmful herbs, and magic arts, from Thessalian lands, that’s his affair” (Kline, 2001, p. 244) – throughout history, plants, natural products of all kinds, charms and rituals were used in the attempt to treat the lovesick patient and to quench the love drive (Ferrand, 1990; Doggett, 2009; McNamara, 2016).

## Objectives and Research Question

Currently there is a debate about the moral implications of producing and using bonding and anti-love drugs such as oxytocin and selective serotonin reuptake inhibitors (Earp and Savulescu, 2018). However, the search for drugs that promote or stop relationships, passionate attraction and lovesickness has remained illusive until relatively recently. Here we try to give a historical overview of the diagnosis and treatment of lovesickness and the herbal and natural product drugs used to influence romantic feelings as well as sexual performance and libido.

In the midlife crisis allegorizing Odyssey (attributed to Homer, ca. 700 BC) Ulysses escapes the transformation into a swine by Circe’s poisoning by taking an antidote. It has been suggested that Circe’s magic consisted of the application of a muscarinic acetylcholine receptor antagonist-containing brew made from deadly nightshade (*Atropa* sp.), mandrake (*Mandragora* sp.), henbane bell (*Scopolia* sp.) or more probably henbane (*Hyoscyamus* sp., all Solanaceae; Plaitakis and Duvoisin, 1983; Müller, 1998; Lee, 1999, 2007). ‘Moly,’ the antidote the Greek god Hermes (*Mercurius*) gives to Ulysses, was proposed to be a species of *Leucojum* or *Galanthus* containing the acetylcholine-esterase inhibitor galanthamine (**2D**), which would be able to counteract the toxic effects of the tropane alkaloids hyoscyamine and scopolamine (**2E**) contained in the Solanaceous drugs (Plaitakis and Duvoisin, 1983; Müller, 1998; Lee, 1999, 2007). When Ulysses was later advised by Circe to take precautions when passing by the island of the sirens, he sealed the ears of his men with bee’s wax he got from Circe and let himself be tied to the mast of the ship in order to escape the enchanting tunes. This suggests that drugs antagonizing erotic attraction were not available in classical antiquity and that Circe was not aware of any remedy protecting from the perils of love. Already Ovid noted: “What use, Medea, to you were herbs of Colchis, when you desired to stay in your father’s house? Circe, what profit to you were Perse’s magic plants when his breeze took Ulysses’ ships away?...You could change men into a thousand shapes, you could not change the commands of your heart” (Kline, 2001, p. 244).

While sealing one’s ears (or eyes) is no solution in a real life setting, we wonder what a woman or a man can do to quench the love passion when no possibility for unification exists. Although Greek mythology is unambiguous, we focus on the questions of whether natural products exist that are able to attenuate the neural circuits involved in the experience of romantic love and whether the accumulated literature over the past 2000 years holds information in this regard. Since neurobiological and pharmacological connections between the love drive and the sex drive exist (*vide infra*), we investigate whether the literature about herbal aphrodisiacs or anaphrodisiacs hold any clues for an anti-love drug. We contextualize our review with neurochemical, ethnomedical and ethnographic data addressing symptoms of lovesickness and methods of diagnosis, as well as the perception of love and lovesickness associated with gender over the centuries (see **Graphical Abstract**).

## Review and Discussion

### Diagnosis of Lovesickness

The origin of psychophysiology and the roots to the psychosomatic approach can be traced back to Erasistratos (early 3rd century BC), Galen (2nd century AD) and Avicenna (Ibn Sina, 980–1037 AD). The Greek physician Erasistratos was not able to diagnose any bodily disease with the sick Antiochus. Only by observing his “stammering speech, fiery flashes, darkened vision, sudden sweats, irregular palpitation of the heart…helplessness, stupor and pallor” whenever his stepmother Stratonice entered the room, Erasistratos deduced the cause of Antiochus’ malady (Mesulam and Perry, 1972). Four centuries later, Galen adopted and refined Erasistratos’ diagnostic approach by observing the pulse rate while simultaneously spelling out the names of different possible love objects (Mesulam and Perry, 1972). Contrary to Erasistratos, who waited for the opportunistic situation, Galen experimented with possible stimuli and analyzed the elicited responses realizing that the name of the love object is enough to trigger the autonomic response (Mesulam and Perry, 1972).

Nine hundred years later, Avicenna treated lovesickness [Avicenna, 2014b; vol. 3, special pathologies, chap. 4: ‘Diseases of the head and their most adverse effects on the senses and conduct’] in the section entitled “On obsession (Scrupulosity): A melancholic disease” (pp. 141–143). The symptoms of ‘obsession,’ as he writes, are sunken and dry eyes, lack of tears when crying, eyelids that come together continuously and fast [probably a dopaminergic effect: see Jongkees and Colzato, 2016], joking and laughing a lot as if he was seeing enjoyable things or as if he would be hearing good news. Moreover, the patient sighs loudly, the breathing is separated and fast and laughter might be interrupted by crying. All organs are moist with exception of the eyes while the eyelids are large and thick from sleeplessness. The pulse is irregular like the pulse of an extremely melancholic person changing from one time to another (Avicenna, 2014b, vol. 3, p. 141).

Avicenna largely follows Galen’s diagnostic approach writing that once the name of the beloved is known she can be called for and a marriage possibly arranged (Hajal, 1994; Avicenna, 2014b, vol. 3, p. 141). According to Avicenna, revealing “the name of the obsession is one of the main treatments for the patient” (vol. 3, p. 141). This initial step of the healing process associated with a confession might be related to the so-called “pathogenic effect of a heavily disturbing secret” on the patient [see Ellenberger (1966) for a thorough discussion and consider customary Catholic practices]. Avicenna explicitly mentions male patients, which suggests that it was socially less accepted to write about women in love or for women to show lovesickness openly. It might also be that women were counseled and attended by female specialists. McNamara (2016), for instance, cites Plato reporting that midwifes acted as matchmakers, suggesting that they were possibly also treating lovesickness.

Examining the pulse was popular for diagnosing lovesickness from the Greco-Roman period until 17th century England (Mesulam and Perry, 1972; Hajal, 1994). McNamara (2016), however, stresses the curiosity that notwithstanding the ancient Greek regarded lovesickness as a true disease, the *Corpus Hippocraticum* does not contain any reference to lovesickness and its treatment. This, he speculates, could be related to Eros having been considered shameful like the ‘sacred disease’ (i.e., epilepsy) and therefore treated by non-Hippocratic healers with symbology, rituals and charms (McNamara, 2016).

Another diagnosis and cultural belief common in the Mediterranean and many West Asian countries associated with psycho-social stress and believed to enter the body through the eyes in a similar fashion as love passions is ‘evil eye’ (Bohigian, 1997; Gross, 1999). Evil eye is said to be a “fateful glance caused by jealousy, envy, or frustration” (Bohigian, 1997). Both evil eye and lovesickness are based on the theory that a beam of light or energy leaving the eye causes the illness (‘extramission’ or ‘emission’ theory; see Gross, 1999 and Págan Cánovas, 2011). Affinities between the love arrow, the arrow of the glance or the envy’s glance described in love poetry [e.g., ‘*De amore*’ (ca. 1186-1190) by Andreas Capellanus or ‘The ring of the dove’ (ca. 1022 AD) by Ibn Hazm] and the evil eye are evident (e.g., Prometheus Bound; Gross, 1999; Spence, 1996). Intriguingly, the symbols used to ward off evil eye, especially on the Italian peninsula, have been associated with phallic and pornographic images since the classic Roman period. The ancient Romans used the ‘*fascinus,*’ a winged phallus deity often represented by bronze effigies (Johns, 1982, p. 69), the raised middle finger (*digitalis infamis*) or “the thumb protruding between the index finger and the next finger” called ‘mano fica,’ which is employed in Italy for rejecting evil eye up to these days (Bohigian, 1997). Above all, the symptoms of evil eye and lovesickness can be very similar (Spence, 1996), which has resulted in misdiagnosed afflictions (Toohey, 2004, p. 134).

### Neurochemistry of Love

Human attraction and romantic love is based on a complex neurobiological network involving altered levels of dopamine, noradrenalin and serotonin (5-HT; **2F**), three neurotransmitters also associated with sexual arousal, motivation and obsessive thinking (Marazziti et al., 1999; Meston and Frohlich, 2000; Fisher, 2004). The caudate nucleus (structure of the dorsal striatum), highly innervated by dopamine neurons, is part of the cerebral reward system and has been shown to be involved in the early and mid-term stage of romantic love by functional magnetic resonance imaging (fMRI; Bartels and Zeki, 2000; Aron et al., 2005). Moreover, the bonding hormones vasopressin and oxytocin, as well as endorphins and the stress hormone cortisol (**2G**) contribute to the process (Esch and Stefano, 2005; Carter and Porges, 2013).

Neural activity of the brain can be visualized by fMRI, which measures the change in blood perfusion in association to the energy required by specific brain areas. Bartels and Zeki (2000) scanned human brains and demonstrated that 2D images of the beloved lead to a unique pattern of activated brain areas comprising the caudate nucleus and the putamen as well as loci in the medial insula and the anterior cingulate cortex. The subjects participating in this study were on average in their 2.4th year of romantic relationship when confronted with pictures of their partners alternated with those of close friends (Bartels and Zeki, 2000). In a similar study conducted by Aron et al. (2005), brains of subjects who had been in love for an average duration of 7.4 months showed an increased activity in the ventral tegmental area (VTA), the right postero-dorsal body and medial caudate nucleus. These studies added evidence to the central importance of dopamine rich areas and showed that the loci of major activity change with progression of romantic love (Bartels and Zeki, 2000; Aron et al., 2005).

Symptom similarities between obsessive–compulsive disorder (OCD) and romantic love such as obsessive and intrusive thinking as well as the involvement of the serotoninergic system in OCD exist (Marazziti et al., 1999; Marazziti and Stahl, 2018). In OCD patients low levels of the 5-HT metabolite 5-hydroxy-indoleacetic acid (**2H**) in the cerebrospinal fluid seems to be associated with low levels of the 5-HT transporter in blood platelets (Marazziti et al., 1999). OCD patients generally respond well to selective serotonin reuptake inhibitors (SSRIs; Brakoulias et al., 2016), which are known to cause sexual dysfunction in both sexes because serotonin generally has an inhibitory function on sexual behavior (Melis and Argiolas, 1995; Rosen et al., 1999). Marazziti et al. (1999) found a significant decrease in 5-HT transporter proteins in the blood platelets of subjects in the state of romantic love similar to that of patients diagnosed with OCD. While a low density of peripheral 5-HT transporters can give no direct information about free circulating or central 5-HT levels, there is evidence that extracellular serotonin in the central nervous system is reflected by plasma serotonin (Sarrias et al., 1990). Based on this assumption Langeslag et al. (2012) investigated the association between plasma and serum serotonin levels, the state of romantic love and obsessive thinking in 20 men and 20 women, 10 of each group being in love for 9 month or less. The intriguing results evidenced gender differences and that the serotonin level of men in love was lower with respect to men not in love and higher in women in love than women not in love (Langeslag et al., 2012). These results merit further investigation since they contradict the hitherto accepted assumption (see e.g., Marazziti et al., 1999; Fisher, 2004). Serotonin is considered to be inhibitory for sexual functioning in both males and females (Melis and Argiolas, 1995; Rosen et al., 1999) and therefore higher serotonin levels in women in love does not make evolutionary sense.

While serotonin plays an inhibitory role, dopamine plays an excitatory role on male sexual behavior (Hull et al., 2004; Bancroft, 2005; Pfaus, 2009). During the post-ejaculatory refractory period in male rats the serotonin level is increased in the lateral hypothalamic area while injecting a serotonin reuptake inhibitor into the same area increases the latency of sexual behavior (Lorrain et al., 1997). The SSRI fluoxetine (**2I**; e.g., Prozac^®^), for instance, leads to decreased sexual appetite in male rats as well as humans (Cantor et al., 1999). The refractory period in humans is more pronounced in males than in females and translating the findings of Langeslag et al. (2012) from the motivation (romantic love) to performance (sexual behavior) one might hypothesize that low central serotonin levels in men during romantic love increases sex drive and performance (reducing the post-ejaculatory interval), which from an evolutionary perspective would make perfect sense.

Cortisol levels were found to be higher in women and men recently (6 month) fallen in love when compared to a control group while testosterone levels in men were decreased and in women increased at early stage relationship (Marazziti and Canale, 2004). At first sight this data stands in contrast with the general view that lust in both sexes is primarily associated with testosterone (**2J**). The decrease in testosterone in men observed by Marazziti and Canale (2004) has, however, hardly any inhibitory effects on sexual performance, as healthy young men have more circulating testosterone than needed for normal sexual functioning (Bancroft, 2005). Marazziti and Canale (2004) interpreted the converging testosterone levels as an evolutionary trick leveling out behavioral differences between men and women securing increased fitness (Marazziti and Canale, 2004). For the theory that increased levels of phenylethylamine (**2K**) are involved in the triggering of romantic love as proposed by Liebowitz (1983), so far no evidence has been found (Marazziti and Canale, 2004). Also the associated story, where the craving for chocolate is linked to its limited amount of non-bioavailable phenylethylamine is a persisting urban legend (Smit et al., 2004; Harvard Health Letter, 2005).

Besides augmented libido and sexual functioning, the lowering serotonin and rising dopamine levels seem to serve the purpose of signaling romantic interest also through the triggering of awkward and ambiguous behavior in the love struck subject while the associated intrusive thinking is necessary for keeping the focus on the love object. Noradrenaline and dopamine are important for the motivation, endurance and energy in courtship behavior and the early phase of the relationship.

### Love Magic

The formulation of love spells and the use of charms with the intent to induce uncontrollable passion are known from different time periods and cultures (e.g., Graeber, 1996; Faraone, 1999; Frankfurter, 2001; Schiltz, 2002; Kang, 2003; Gutiérrez, 2007; Musharbash, 2010; van Andel et al., 2015), and it appears that in classical antiquity and the late Roman world the authors of erotic spells were just as much men as women (Dickie, 2000). However, in one of the darkest chapters of European history Kramer and Sprenger (1928) in their infamous *Malleus maleficarum* (‘Hexenhammer’; part II, question II, Chap. III) write: “Philocaption, or inordinate love of one person for another, can be caused in three ways. Sometimes it is due merely to a lack of control over the eyes; sometimes to the temptation of devils; sometimes to the spells of necromancers and witches, with the help of devils.” In the section treating the question “whether witches can sway the minds of men to love or hatred” (part l, question VII) the witch hunters ask: “Is it a Catholic view to maintain that witches can infect the minds of men with an inordinate love of strange women, and so inflame their hearts that by no shame or punishment, by no words or actions can they be forced to desist from such love; and that similarly they can stir up such hatred between married couples that they are unable in any way to perform the procreant functions of marriage; so that, indeed, in the untimely silence of night, they cover great distances in search of mistresses and irregular lovers?” (Kramer and Sprenger, 1928).

**FIGURE 1:**
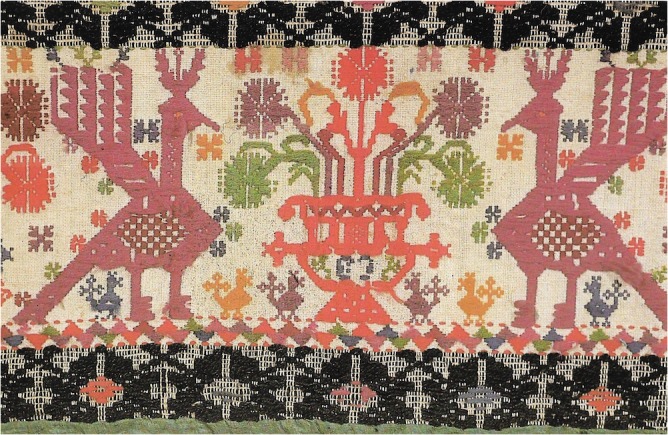
Sardinian (Italy) handicraft showing fertility and love symbols.

Apple-like fruits (fruit of temptation in the Garden of Eden) such as apple (*Malus*, sp.), quince (*Cydonia oblonga* Mill., Rosaceae) and pomegranate (*Punica granatum* L., Lythraceae) as well as other fruits containing small seeds, played a role as love symbols during seduction and courtship behavior in the Mediterranean (Taberner, 1985, p. 52; Faraone, 1999, pp. 69–71). The pomegranate flanked by turtle doves (*Streptopelia turtur* L.) is a motive symbolizing fertility and love and is still used in handicraft in Sardinia today (**Figure 1**).

**FIGURE 2:**
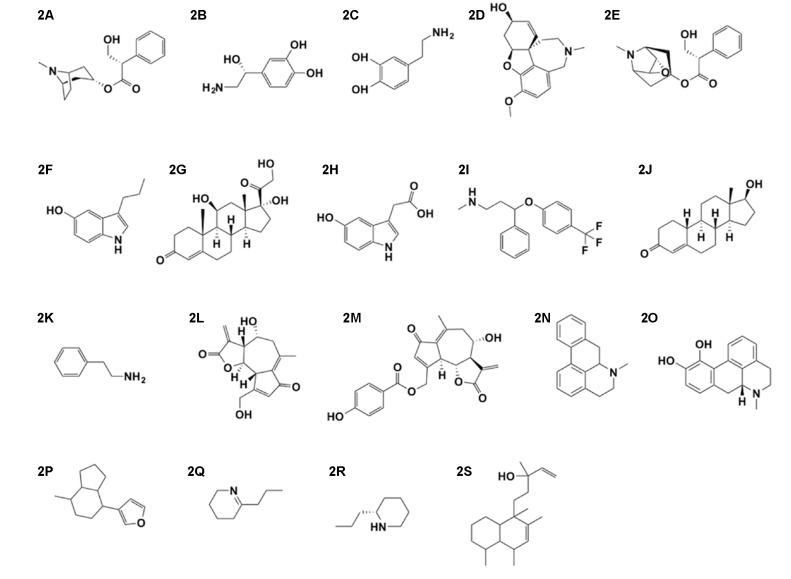
**(A)** L-hyoscyamine, **(B)** noradrenalin, **(C)** dopamine, **(D)** galanthamine, **(E)** scopolamine, **(F)** serotonin, **(G)** cortisol, **(H)** 5-hydroxy-indoleacetic acid, **(I)** fluoxetine, **(J)** testosterone, **(K)** phenylethylamine, **(L)** lactucin, **(M)** lactucopicrin, **(N)** aporphine, **(O)** apomorphine, **(P)** 5-(3-furyl)-8-methyl-octahydroindolizine, **(Q)** γ-coniceine, **(R)** coniine, and **(S)** clerodadienol.

**FIGURE 3:**
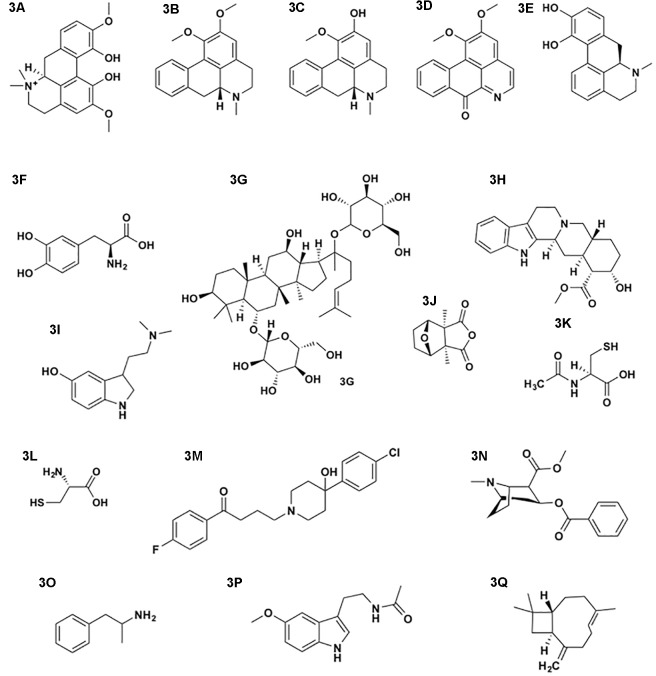
**(A)** magnoflorine, **(B)** nuciferine, **(C)** asimilobine, **(D)** lysicamine, **(E)** apomorphine, **(F)** L-DOPA, **(G)** ginsenoside, **(H)** yohimbine, **(I)** bufotenine, **(J)** cantharidin, **(K)** *N*-acetylcysteine, **(L)** cysteine, **(M)** haloperidol, **(N)** cocaine, **(O)** amphetamine, **(P)** melatonin, and **(Q)** (E)-β-caryophyllene.

The faith in the effectiveness of love potions (also ‘philters,’ see e.g., Anonymous, 1909) was very common and, according to Birchler (1975), not even Paracelsus (1493–1541) was immune against the belief that lovesickness would have its origin in a love potion while Lewin (1920, p. 20) noted that toward the end of the 18th century the common law of Prussia contained a special paragraph treating the administration of love potions. Besides the inclusion of stimulating and intoxicating substances, a lot of ingredients were based on magical beliefs such as the organs and blood of different animals but above all substances deriving from the lovesick object (Lewin, 1920, p. 16–18). These included excretes and secretes, which were regarded as being toxic by many medical authors throughout history such as menstrual blood, mucus, excrement, placenta and the umbilical cord (Birchler, 1975). Also hair and skin were believed to contain the ‘vital spirit’ (Watt, 2010). Already Pliny the older (23–79 AD) provides evidence for the widely regarded belief in the toxicity of menstrual blood (Birchler, 1975). Some scholars thought that the menstrual blood of women in love would contain a love virus (‘*Virus amatorium’*; Birchler, 1975) while menstrual blood was regarded as a sort of female semen (Watt, 2010). Lewin (1920, pp. 16–17) disenchants the idea surrounding the effectiveness of love potions (*pocula amatoria*), which, as he writes, are to be classified as aphrodisiacs (*pocula libidinis*): It is impossible to provoke feelings of affection with such means; rather the sober mind is being nocked out, and the functioning of the central nervous system deranged in such a way that libido is enhanced or have an irritating effect on the genitals (Lewin, 1920, p. 17).

### Romantic Love, Sex and Associated Gender Issues in Europe and the Mediterranean World

Eros is the illegitimate son of the beauty and love goddess Aphrodite (*Venus*) and the war god Ares (*Mars*). In the ancient Greek world Eros was present in marriage only for securing the procreation of legitimate children and future citizens (Cantarella, 2009, p. 11). Passionate love was experienced in different contexts as the origin of Eros indicates (Cantarella, 2009, p. 11). Plato suggests that the upper class of Greek society followed a rather rational and practical approach toward romantic love: Once women and men have exceeded the age of procreation we allow them to make love with whom they want, recommending warmly to not let see any fruit of the womb the light of the day in case it should form (Lewin, 1920, p. 15 and references therein). The array of herbal drugs that were used as abortifacients throughout written history and the legal practices adopted by different cultures for making abortion punishable are reviewed in Lewin and Brenning (1899) and Riddle (1994). Those who were sentenced for inducing abortion were, of course, women, particularly midwifes but also the mothers themselves (Leibrock-Plehn, 1992).

The role of the poisoner in Greek and Roman mythology is generally cast by female figures such as Circe and Medea or Canidia and Sagana. Medea was Circe’s niece and daughter of king Aeëtes, ruler over the magic lands of Colchis. There, the barbarian princess served as a priestess of Hecate, who was the goddess of magic and witchcraft as well as mistress of the deadly and healing plants growing in Colchis (Lewin, 1920, pp. 1–3; Toohey, 2004, p. 59–103). The preparation of love potions was regarded a women’s domain (especially that of prostitutes and witches) since the classic period (Birchler, 1975) and similarly also the women accused of witchcraft during the Renaissance period were said to prepare intoxicating and psychotropic ointments and potions (Kramer and Sprenger, 1928; Müller, 1998; Ginzburg, 1990; Piomelli and Pollio, 1994). On top of that the body of women was believed to be the site of production of toxic substances itself (see Birchler, 1975).

In classical antiquity and the Middle Ages lovesickness was perceived as a malady affecting both sexes (Kline, 2001; Capellanus, 2006; Avicenna, 2014b, vol. 3, pp. 141–143; McNamara, 2016) while according to Birchler (1975) with the beginning of the Age of Enlightenment (ca. 1650) lovesickness was gradually becoming a gynecological disease. This medical view lasted until the beginning of the 19th century and the ailment was commonly referred to as hysteria (Birchler, 1975).

As Avicenna (2014b, vol. 3, p. 143) testifies, women served as a remedy for men in case of lovesickness: “one of the helpful entertainments for a male patient is to buy many slave girls and have him have sexual intercourse with them. He should have parties with them” while recommendations for inverse constellations are described only very rarely in the literature. Birchler (1975) cites a German reference from 1717 saying that as a cure [for lovesickness] nothing helps better and faster than cohabitation with a good and strong fellow.

While the Greek and Roman Pantheon were populated with numerous venerable deities a monotheist system offers no choice: When seduced by the serpent, Eve and then Adam, encouraged by Eve, tasted the forbidden fruit of knowledge of good and evil and recognized their love and sexual attraction for each other, they were readily expelled from paradise. Having lost eternal life, Adam was condemned to hard labor on the fields and Eve to the pain of childbearing and to subduing herself to Adam, securing the procreation of humankind^2^. However, similar to Eve, also Pandora – the ‘Greek Eve’ – is hold responsible for all calamities afflicting human societies and represents the damnation of humanity (Cantarella, 2009, p. 133–135).

Consequently, women were regarded as the producers of love potions, easily corruptible by seductive and malignant forces such as the devil and therefore as *the* source of erotic attraction and lovesickness (see Kramer and Sprenger, 1928). Kramer and Sprenger (1928) conclude that “all witchcraft comes from carnal lust, which is in women insatiable,” which according to Tennov (1998, p. 236) testifies to the theory that the *Malleus* was not just a guide for identifying witches but also “an explicit statement that women as man’s love objects is the cause of all evil.” The *Malleus maleficarum* is in fact a bloody-minded and misogynous continuation of the tale of Adam and Eve (which is misogynous in itself). In essence, it portrays the psychology of men having difficulties in controlling their sexual instinct and maintaining a monogamous relationship but assigning the guilt exclusively to women. Islamic cultures take a preventive approach toward the contraction of lovesickness and evil eye altogether by obliging women to cover their head and chest with a veil in the presence of men not part of their close family.

Evidently in human societies around the Mediterranean the cultural expressions and implications of love, sex and procreation are organized along hierarchical lines that put men on top since antiquity.

### Natural Product Drugs, Regimen and Cures Traditionally Used for Treating Lovesickness

In ‘*Remedia Amoris*’ Ovid writes (ca. 1–2 AD): “If you’ve regrets, and moderate emotions touch your heart, then halt your feet, while you can... Halt its beginnings: it’s too late for the doctor to be called, when the illness has grown stronger through delay. Either you try, if you can, to quench a fire at the start, or when it dies down, through its own violence.” When there is no possibility for union, Ovid and Avicenna recommended different social behavioral approaches for overcoming lovesickness. Ovid suggested to contemplate the defects of the love object (Kline, 2001, p. 247) while Avicenna (2014b, vol. 3, p. 142) and others advised to have an older (and particularly ugly looking; see Birchler, 1975) women saying bad and negative things about the love object, which then should be repeated by the patient.

Apart from receiving good food the patient should be distracted, kept busy by having arguments and conversations with other people, going hunting or engage in different activities (Avicenna, 2014b, vol. 3, p. 142). Ovid gives cause for concern: “Venus loves idleness: you who seek to end love, love gives way to business: be busy, you’ll be safe. That Boy’s [Amor] accustomed to following idleness: he hates the busy: give your vacant mind work to occupy it” (Kline, 2001, pp. 239–240). Ovid further suggested: “You who love, beware lonely places, lonely places are harmful! Why flee? You can be safer in a crowd. You don’t need secrecy (secrecy nurtures passion): in future it’s the crowd that will assist you” (Kline, 2001, p. 257).

Common practices for healing lovesickness in classical antiquity seem to have been purification ceremonies where the clothes of the love object were fumigated [e.g., with sulfur (see also Kline, 2001, p. 244) and salt] with the concomitant muttering of incantations (Hordern, 2002). Such practices potentially mediated a placebo effect, i.e., a response of its meaning to the afflicted person (see also the ‘meaning response’ in Moerman and Jonas, 2002).

Regarding the use of herbs for amatory attraction, Ovid is pretty sharp (see Introduction), which does not mean that he did not believe in libido enhancing properties of plants. In fact, Ovid recommended to specifically abstain from Italian onions, onions from the Libyan shores as well as those from the Greek Megara, as these would be harmful (aphrodisiacs). Probably besides varieties of *Allium cepa* L. also *Leopoldia comosa* (L.) Parl. is addressed in his warnings. He further advised to avoid garden rocket [*Eruca vesicaria* (L.) Cav., and *Diplotaxis tenuifolia* (L.) DC., Brassicaceae] and anything else that prepares the human body for making love (see below). Instead, he recommended eating rue (*Ruta* sp., Rutaceae), “which sharpens the eyesight” [for better contemplating her/his defects?] and whatever prevents from making love. Herbs used to calm love melancholy and libido were generally described as having cooling properties (Ferrand, 1990). Avicenna (2014b, vol. 3, p. 143) furthermore recommends the longer “medical paste antidote” for cleansing the patient^3^. This complex composition contains sedative (balm leaves and seeds, valerian, and opium) as well as excitatory drugs (rocket seeds, rock parsley).

It seems that the biphasic effect of alcohol has been well recognized as Ovid noted that “wine prepares your heart for love, unless you take enough, and your wits are stupefied, overcome by the neat juice. So don’t drink at all, or drink so much your cares all vanish: if it’s anywhere between the two it’s bound to do you harm” (Kline, 2001, p. 266). According to Preuss (1971, p. 538) drinking wine in the classical Roman as well as Jewish culture was a privilege of men who feared that the pro-social and aphrodisiac effects of alcohol would provoke their wives and daughters to commit adultery or becoming frivolous (Preuss, 1971, p. 538).

### Anaphrodisiacs

Several drugs claimed to prevent libidinous dreams and intercourse were mentioned by Dioscorides (1st century AD) in *De Materia Medica* (*DMM*) as for instance the seeds of *Lactuca sativa* L. and *L. serriola* L. (Matthioli, 1568, p. 549, Lattuca domestica and selvatica, Asteraceae). Lactucarium, the condensed latex of lettuce, was used since ancient times to falsify opium (Madaus, 1987, p. 1701). *L. sativa* as well as *L. virosa* contain the guaianolide sesquiterpene lactones lactucin (**2L**) and lactucopicrin (**2M**; Wesołowska et al., 2006; Chadwick et al., 2013). Both compounds showed analgesic and sedative effects in mice in the hot plate (15 and 30 mg/kg) and in the spontaneous locomotor activity test (at 30 mg/kg), respectively (Wesołowska et al., 2006). These data suggest that the alleged anaphrodisiac effects might be due to the narcotic properties of these guaianolides.

The seeds and roots of the water lily (*Nymphaea alba* L., Nimphea, Nyphaeaceae) are said to be useful “against becoming perverted during the dreams at night” (Matthioli, 1568, p. 843). However, the literature about the neuropharmacological and excitatory effects of *Nymphaea* species is contradictory while the chemistry is fragmentary. From *N. alba* the nuphar alkaloids (quinolizidines) nupharine and nympheine (structures unknown) were obtained (Wróbel, 1967; Emboden, 1981) while *Nymphaea ampla* (Salisb.) DC. afforded aporphine (**2N**), a compound closely related to the dopamine agonist apomorphine (**2O**), which provides supporting evidence for the use of *N. ampla* and *Nymphaea nouchali* var. *caerulea* (Savigny) Verdc. as entheogens and aphrodisiacs (Emboden, 1981; Bertol et al., 2004). Nuphar alkaloids are also a component of *castoreum*, the secretion obtained from the castor sacs of beavers (*Castor* sp.), used by the animals to mark their territory (Maurer and Ohloff, 1976). In succession of a chemical study aimed at the elucidation of the stereochemistry of a minor *castoreum* alkaloid, Seki and Georg (2014) found 5-(3-furyl)-8-methyl-octahydroindolizine (**2P**) to interact with the oxytocin receptor (*K*_*i*_ = 0.6 μM) suggesting that other nuphar alkaloids might also interact and that similar to the biological roles of oxytocin these alkaloids are used in social communication by beavers. The traditional uses of *castoreum* including the promotion of menstruation, the expulsion of the placenta and the fetus might well be related to such an interaction (Matthioli, 1568, p. 352; Seki and Georg, 2014).

Also the seeds and leaves of *Conium maculatum* L. (Matthioli, 1568, p. 1154–1155; Cicuta, Apiaceae) applied externally were recommended by Dioscorides to prevent sexual dreams. *Succus conii* (juice of hemlock) was part of the British Pharmacopoeia and the British Pharmaceutical Codex until 1934 and used as a sedative and antispasmodic. The effects of hemlock poisoning such as weakness, tremor, paralysis and respiratory failure are due to the neurotoxic properties of γ-coniceine (**2Q**) and coniine (**2R**), mediated via the nicotinic receptor (nAChR; Reynolds, 2005; Schep et al., 2009).

One of the most famous herbal anaphrodisiacs is the chaste tree (*Vitex agnus-castus* L., Lamiaceae). About *V. agnus-castus* Dioscorides writes (Matthioli, 1568, p. 213, Vitice) that the seeds, when drunk, would make the milk flow and dry up the sperm, adding that the ancient Greek called this tree ‘agnos’ (i.e., ‘*casto*’) because the women who in relation to the sacrifices for *Ceres* tried to remain chaste prepared their beds with the leaves of this tree (Matthioli, 1568, p. 213). Similarly, Avicenna (2012, vol. 2, p. 228) mentions that to “prevent nocturnal emission and sexual excitement” the “branches are spread on the back of a person” and that leaves taken orally dry semen. Today, chaste tree seed extracts are used to treat premenstrual syndromes such as premenstrual mastalgia for which hyperprolactinemia seems to be the underlying factor (Wuttke et al., 2003). Men and women with increased levels of prolactin often report a decrease in sexual interest, which can be treated with agonists of the dopamine receptor (Meston and Frohlich, 2000). It appears that *V. agnus-castus* would rather mediate aphrodisiac effects as Wuttke et al. (2003) summarize that *V. agnus-castus* seeds contain clerodane type diterpenes (clerodadienols e.g., **2S**) with dopaminergic activity at the D_2_ receptor *in vitro*, able to suppress the release of prolactin in cultivated pituitary cells. However, a 14 days placebo-controlled clinical study involving 20 healthy men found increased serum prolactin levels associated with a lower dose (120 mg/day) of *agnus castus* extract and decreased prolactin levels with a higher dose (480 mg/day; Merz et al., 1996). The sexual desire reducing property of purslane (*Portulaca oleracea* L., Portulcaceae) described in *DMM* (Matthioli, 1568, p. 503, Portulaca) seems to lack any pharmacological basis so far (Iranshahy et al., 2017). Regarding the libido modulating properties of *Mentha* sp. (Lamiaceae) Dioscorides (Matthioli, 1568, p. 749, Menta, Lamiaceae) and Avicenna (2014b, vol. 3, p. 1196) disagree. While Dioscorides mentions the libido enhancing properties of the herb, Avicenna recommends it together with other drugs as a treatment for reducing sexual desire. The fact that customary mint tea (infusion) consumption by men of two small Turkish towns was made responsible for the experienced decreased libido (Akdogan et al., 2004) lends credits to Avicenna’s notions. Moreover, after receiving *Mentha spicata* L. and *M. x piperita* L. infusion ad libitum for 30 days, rats showed significant and dose dependent decrease of plasma testosterone when compared to a control group (Akdogan et al., 2004). Avicenna (2014b, vol. 3, pp. 1194–1195) further recommended cooling drugs such as lettuce, purslane, endivie (*Cichorium* sp., Asteraceae), zucchini (*Cucurbita pepo* L.) squirting cucumber (*Ecballium elaterium* (L.) A.Rich.; both Cucurbitaceae), coriander leaves (*Coriandrum sativum* L., Apiaceae), poultice of water lily, sour cherry (*Prunus cerasus* L., Rosaceae) and cold tempered fruits as well as poultices made from cold tempered oils in general and to rub camphor on the sexual organs.

Several herbal anaphrodisiacs and aphrodisiacs (*vide infra*) show a continued appearance in the herbal texts over the centuries also because the written sources served as blueprints for the transmission of medical knowledge (Leonti, 2011).

### Aphrodisiacs

Herbal remedies and natural products used to increase libido, potency and sexual pleasure are known since ancient times from cultures across the globe (Taberner, 1985; Cheikh Nefzaoi, 1886; Sandroni, 2001). Today such drugs are also advertised in popular non-fiction books (e.g., Rätsch, 1995). As a first line treatment of sexual dysfunction and for improving libido, however, food and spices were of central importance (Avicenna, 2014b, vol. 3, pp. 1177–1194). Spices and fruits, especially when exotic, imported from elsewhere and rare were likely to be used as aphrodisiacs (e.g., tomato and potato; Taberner, 1985, pp. 60–61). Also the particular shape of a herbal drug along with the doctrine of signatures often directed its use as an aphrodisiac [e.g., roots of *Orchis* sp., (Orchidaceae) resembling testicles or the phallic and anthropomorphic roots of *Arum maculatum* L. (Araceae) and *Panax ginseng* C.A.Mey (Araliaceae); Taberner, 1985].

Unfortunately, the passage on aphrodisiacs in Theophrastus’ ‘Enquiry into Plants,’ together with that on drugs for potency and fertility, has been excluded by the editor from the 1916 Loeb version of the text (McNamara, 2016). Among the aphrodisiac drugs reported by Theophrastus was mandrake root (*Mandragora* spp., Solanaceae) and bulbs of cyclamen (*Cyclamen* spp., Primulaceae; McNamara, 2016). Both drugs are also mentioned in relation to love magic (‘in cose amatorie’) by Dioscorides [Matthioli, 1568, p. 1132–1134 (Mandragora) and p. 620 (Ciclamino)].

The fruits of the mandrake were called ‘apples of love’ and the plant associated with both, Aphrodite, the Greek love goddess as well as Circe, the sorceress (Lee, 2006; Kennedy, 2014, p. 132). The fruit of ‘dudaim’ (book Genesis), which Lea handed over to her sister Rahel upon request, and brought temporarily Jakob as well as pregnancy back to Lea, is generally identified as a mandrake ‘apple’ (Hanuš et al., 2005; Lee, 2006; Chidiac et al., 2012) but an unambiguous identification is probably impossible (Preuss, 1971, p. 539–540). The mandrake root was used as an anesthetic, as an aphrodisiac and fertility drug, for amatory attraction and magic until the 17th century (Lee, 2006; Chidiac et al., 2012). The whole plant contains intoxicating tropane alkaloids, which produce the typical effects of muscarinic acetylcholine receptor antagonists but seems to be phytochemically more complex than *Atropa* spp., *Hyoscyamus* spp. or *Datura* spp. (see Hanuš et al., 2005).

Dioscorides recommended the leaves of *Allium ampeloprasum* L. (Matthioli, 1568, p. 579, Porro capitato, Alliaceae) and *Allium* sp. (Matthioli, 1568, p. 592, Scordopraso, Alliaceae) as aphrodisiacs (‘stimolare venere’). Semitic and non-Semitic tribes in the Near East considered garlic (*Allium sativum* L., Alliaceae) as a powerful aphrodisiac able to increase sperm (Preuss, 1971, p. 538). Other monocots mentioned in *DMM* as aphrodisiacs (‘awaken venereal appetite’) are the edible corms of *Leopoldia comosa* (L.) Parl. (Matthioli, 1568, p. 635, Bulbo che si mangia, Asparagaceae) and the bulbs of *Gladiolus italicus* Mill. (Matthioli, 1568, p. 1041, Xiphio, Iridaceae).

Generally drugs with ascribed aphrodisiac properties in *DMM* were also frequently used as food, including several members of the Brassicaceae such as the seeds and roots of *Brassica rapa* L. (Matthioli, 1568, p.: 460, rape), seeds and leaves of *E. vesicaria* and *D. tenuifolia* (Matthioli, 1568, p. 559–560, Ruchetta) as well as the seeds and leaves of *Nasturtium officinale* R.Br. (Matthioli, 1568, p. 596, Nasturtio). While the Brassicaceae seeds are rich in fatty acids (Yaniv et al., 1998), the aerial parts of these species are rich in glucosinolates and glucosidated flavonoids (Mithen, 2006; Bell and Wagstaff, 2014).

Other examples are the seeds of *Pimpinella anisum* L. (Matthioli, 1568, p. 796, Aniso, Apiaceae) to strengthen sexual intercourse, carrots (Matthioli, 1568, p. 789, *Daucus carota* L., Pastinaca, Apiaceae) or the tubers of *Arum* sp. (Matthioli, 1568, p. 628, Aro, Araceae) said to ignite venereal appetite, as well as the tubers of *Dracunculus vulgaris* Schott (Matthioli, 1568, p. 622, Dragontea maggiore and minore, Araceae). According to Koerper and Kolls (1999) literary and coinage contexts suggest that the extinct *Silphium* (probably a *Ferula* species, Apiaceae) was also a treasured aphrodisiac.

Two multi-ingredient Amrita (Soma) recipes produced in Kashmir and preserved in the 6th century ‘Bower Manuscript’ are said to increase the strength of men and produce “loveliness and grace in women,” predisposing them for conception (Hoernle, 2011; Leonti and Casu, 2014). Two important ingredients (*Tinospora cordifolia* (Willd.) Hook.f. & Thomson, Menispermaceae and *Nelumbo nucifera* Gaertn., Nelumbonaceae) contain aporphine type alkaloids such as magnoflorine (**3A**), nuciferine (**3B**), asimilobine (**3C**) and lysicamine (**3D**; Leonti and Casu, 2014), which are closely related to apomorphine (**3E**), a drug used to treat erectile dysfunction (Ixense or Uprima). Apomorphine is an agonist at the dopamine D_2_ receptor and to a lesser extent at the D_1_ receptor due to its structural similarities with dopamine and acts as a central initiator of erection (Altwein and Keuler, 2001). However, the ingredients most frequently mentioned in the aphrodisiac recipes (clarified butter) reported in the Bower Manuscript are: (root) juice of *Ipomoea cheirophylla* O’Donell, (Convolvulaceae), seeds of cowhage [*Mucuna pruriens* (L.) DC., Fabaceae], *Vigna mungo* (L.) Hepper. (Fabaceae), fruits of long pepper (*Piper longum* L., Piperaceae), fruits of emblic myrobalan (*Phyllanthus emblica* L., Phyllanthaceae) and fruits of *Tribulus terrestris* L. (Zygophyllaceae; Hoernle, 2011). While the seeds of *M. pruriens* contain high amounts of L-DOPA (**3F**), for producing a central effect they would need to be administered together with a DOPA decarboxylase inhibitor in order to prevent L-DOPA’s peripheral conversion into dopamine. The existing phytochemical and pharmacological data regarding the alleged aphrodisiac properties of *T. terrestris* are inconclusive (Neychev and Mitev, 2016). For the remaining taxa no data or evidence sustaining any libido enhancing activity could be found.

A widely used herbal medicine for treating erectile dysfunction in Asian countries is the root of *Panax ginseng* C.A. Mey. (Araliaceae). Pharmacological evidence suggests that ginseng and ginsenosides (**3G**) act through central (anxiolytic action and enhancing striatal dopamine activity) as well as peripheral (NO release) mechanisms (Murphy and Lee, 2002). Scientific evidence supporting the widespread use of *Turnera diffusa* L. (Passifloraceae) as an aphrodisiac is limited and proper clinical trials are lacking (Szewczyk and Zidorn, 2014).

Yohimbine (**3H**), present in the leaves and the bark of the West African Yohimbe tree *Pausinystalia johimbe* (K. Schum.) Pierre ex Beille (Rubiaceae), is mainly used against erectile dysfunction. Yohimbine is a highly potent antagonist at the α2-adrenorecpetor resulting in an increase in sympathetic tone and blood pressure but the exact mechanism by which the compound increases sexual functioning is not completely elucidated (Tam et al., 2001).

Animal derived products used as aphrodisiacs across cultures and history include *Ambra grisea* deriving from the gut of the sperm whale (*Physeter macrocephalus* L.), which is also used in perfumery on the Arabic peninsula, the psychoactive bufotenine (**3I**) containing skin of *Bufo* spp. (Bufonidae) in China and the toxic cantharidin (**3J**) from the Spanish fly (*Lytta vesicatoria* L., Meloidae) native to Europe (Sandroni, 2001). Besides the particular organoleptic properties, oysters (Ostreidae) contain exceptional high levels of zinc (Murphy et al., 1975). Zinc deficiency in humans has been shown to cause infertility through inhibition of spermatogenesis and decrease of testosterone levels (Prasad, 1991; Hunt et al., 1992; Bedwal and Bahuguna, 1994). The concentration of zinc in the seminal plasma is positively associated with the quality of sperm (Colagar et al., 2009).

### Modern Approaches for Fighting Lovesickness

Different approaches to overcome lovesickness from Ovid (1st century AD) to Fisher (2004) have been proposed. These were mostly social behaviors, which should distract from the agony of unrequited love including the avoidance of the love object, physical activity, engaging in ‘new’ pastimes and, especially advised in the older literature, to act out the associated sex drive (Ferrand, 1990; Tennov, 1998; Kline, 2001; Fisher, 2004).

Insights gained from neurobiological studies, however, offer possibilities for direct or indirect pharmacological treatments in order to stabilize and normalize brain chemistry (Fisher, 2004; Young, 2009; Earp et al., 2013). Today, instead of love potions, the attachment or ‘love’ hormone oxytocin (e.g., OxyLuv, Oxytocin Nasal Spray), a pro-social neuropeptide, is advertised in order to preserve or restore affectionate bonding between (married) couples. Intranasal oxytocin influences men living in a monogamous relationship in such a way, that they avoid attractive women in a first encounter, whereas the neuropeptide has no such effect on men not being in a relationship (Scheele et al., 2012). In a placebo controlled fMRI study conducted by Scheele et al. (2013) men subjects rated their female partner’s face as being more attractive when compared to other women when receiving intranasal oxytocin beforehand. However, the effects of oxytocin seem to depend on the context of the specific situation as well as personal characteristics (Bartz et al., 2011).

#### Drugs Interacting With the Serotoninergic and Glutamatergic System

It has been shown that serotonin production in the brain is directly correlated with sunlight exposure (Lambert et al., 2002). Since a low serum and plasma serotonin level is associated with romantic love in men (Langeslag et al., 2012), exposing oneself to sunlight may help to restore normal serotonin levels. As already noted by Fisher (2004), antidepressants such as SSRIs could be used to adjust serotonin levels and treat obsessive thinking in parallel to the standard treatment of OCD (Brakoulias et al., 2016). SSRIs are prescription drugs, and while not all OCD patients respond to SSRIs, these compounds can cause sexual dysfunction (Crenshaw and Goldberg, 1996) and presumably diminish the probability of falling (happily) in love (Fisher, 2004). Accumulating evidence suggests that also the glutamatergic system is involved in the symptomatology of OCD offering new possibilities for treatment (Kariuki-Nyuthe et al., 2014; Marazziti et al., 2018). The prodrug *N*-acetylcysteine (**3K**; NAC) is classified as a nutraceutical and was first used for its mucolytic properties while the derived cysteine serves to restore depleted glutathione (GSH) reserves in the liver (Rushworth and Megson, 2014). GSH depletion is also a common feature in a range of neuropsychiatric disorders. Cysteine (**3L**) increases extracellular glutamate levels, which leads to decreased neurotransmitter release. NAC has been found to have positive effects in a range of neuropsychiatric disorders, such as schizophrenia, addiction, OCD (only one case report) as well as bipolar disorders (Rushworth and Megson, 2014; Marazziti et al., 2018).

#### Drugs Interacting With the Dopaminergic System

The involvement of dopamine rich areas, including the VTA, in the brains of love struck subjects has been shown through fMRI studies by Bartels and Zeki (2000) as well as Aron et al. (2005). Romantic love shows many psychological, behavioral and biochemical characteristics that are also common in addiction. These include focused attention, “mood swings, craving, obsession, compulsion, distortion of reality, emotional dependence, personality changes, risk-taking, and loss of self-control,” and are mediated by the mesolimbic reward circuit through the release of dopamine (Abbott, 2002; Fisher et al., 2010). Consequently, it has been argued that research investigating romantic love and substance abuse could inform each other (Fisher et al., 2016). Since dopamine is thought to be one of the main neurotransmitters involved in the experience of romantic love as well as addiction, a system able to attenuate dopaminergic firing might bring some relieve. Antipsychotic dopamine antagonists (e.g., haloperidol, **3M**) are already in use for the treatment of alcohol abuse and as antidotes for dopaminergic drugs such as cocaine (**3N**) and amphetamine (**3O**). These are, however, prescription drugs that can have serious adverse effects and, since lovesickness is not regarded a disease according to the official disease classifications (see Introduction), not a politically or ethically justified treatment.

Melatonin (**3P**) mediates anti-dopaminergic effect in the striatum (Zisapel, 2001). Neural activity in the caudate-putamen region is inhibited upon melatonin as well as vasotocin treatment in pinealectomized rats (Castillo-Romero et al., 1993). Shoja et al. (2007) therefore hypothesize that the pineal gland products might be used to cure infatuation and attenuate romantic feelings.

Recently, however, the endocannabinoid system has come into focus as a target for the treatment of addiction (Sloan et al., 2017). Intriguing results were obtained with selective CB_2_ receptor agonists, which were shown to inhibit cocaine self-administration in wild-type mice but not in CB_2_ knock out mice (Xi et al., 2011; Morales and Bonci, 2012). A more recent study by Zhang et al. (2014) reports that the inhibitory effect on cocaine self-administration is mediated by CB_2_ receptors present on dopaminergic neurons of the VTA through inhibition of dopaminergic firing. The sesquiterpene (*E*)-*β*-caryophyllene (**3Q**; BCP) is an essential oil component present across plant phylogeny and a natural dietary product (also approved as a food additive by the FDA) adding to the flavor of a wide range of spices and herbs. This volatile and lipophilic compound has been shown to selectively bind to the CB_2_ receptor (*K*_*i*_ = 155 nM) acting as a functional agonist and mediating anti-inflammatory effects (Gertsch et al., 2008). BCP has been shown to pass the blood brain barrier (Varga et al., 2018) and to dose-dependently attenuate the consumption and preference of alcohol in mice, an effect reversible with previous administration of a CB_2_ receptor antagonist (Al Mansouri et al., 2014).

CB_2_ receptors expressed on dopaminergic neurons of the VTA, part of the brain’s reward circuit implicated with addiction, orgasm and strong emotions such as love, might constitute a new pharmacologic target. Bioavailable CB_2_ agonists such as the common food additive *β*-caryophyllene might have the potential to attenuate dopaminergic firing and to quench the reward and thus motivation associated with romantic love.

## Conclusion

As human biology is reflected in cultural expression, the experience of romantic love and unrequited erotic attraction can be traced through philosophy, mythology, medicine, literature and art in general. The management and expressions of love, sex and reproduction have been shaped by the economic constraints of sedentary agriculture resulting in an economization of romantic love. A deep moral discrepancy regarding the recommendation of engaging in sexual intercourse as a remedy for lovesickness between the medical and clerical authorities must have existed throughout the ages.

Similar to the literature and folklore where recipes for inducing mutual passion are far more abundant than for overcoming lovesickness, the literature dealing with aphrodisiacs is far more prolific with respect to that treating anaphrodisiacs. This is not unexpected as drugs with the ability to decrease sexual desire were culturally relevant only during and while preparing for ceremonial and religious practices. Often the herbal drugs used as aphrodisiacs and anaphrodisiacs were also food items consumed at a regular basis. It was probably the dietary use that allowed people to detect the libido modulating effects. The pharmacological modes of action of aphrodisiacs and anaphrodisiacs are heterogeneous and divers. Besides sedative and toxic effects the libido dampening properties seem to be associated with decreasing testosterone levels (*Mentha* spp.) while for *Nymphaea* spp. and *Vitex agnus-castus* the chemical and pharmacological data are ambiguous and do not permit any conclusions to be drawn about their alleged anaphrodisiac properties. Especially for the selection of aphrodisiacs organoleptic cues appear to have been important. Their properties and mode of actions range from phallus and testicle-alluding shapes and spicy tastes (e.g., *Allium* spp. and different Brassicaceae taxa) to intoxicating effects (Solanaceous drugs), dopaminergic (apomorphine type alkaloids) and adrenergic (yohimbine) interactions as well as peripheral NO release (*Panax* spp.).

Ovid was correct in not trusting the properties of herbs for love magic and directed his recommendations toward a behavioral approach and social regime. Insights into neurobiology together with chemistry and pharmacology, however, are opening completely new avenues into chemical approaches toward lovesickness. For treating the addictive component and intrusive thinking associated with lovesickness one could experiment with the food additive *β*-caryophyllene and the nutraceutical *N*-acetylcysteine.

Little is actually known about the neurobiological predispositions necessary for falling in love and conflicting data regarding the up and down regulation of involved neurotransmitters during the early stage of romantic love exist. As it is yet impossible to anticipate the ‘magic moment’ and prepare for an fMRI study and as it is not possible to conduct brain microdialysis in humans, the exact neuronal activities associated with falling in love will probably remain forever nature’s and Eros’ secret.

“He did what he could, he loved: better than doing nothing” (Ovid – *Remedia Amoris*)

## Author Contributions

ML wrote the paper together with LC and both approved it.

## Conflict of Interest Statement

The authors declare that the research was conducted in the absence of any commercial or financial relationships that could be construed as a potential conflict of interest.

## Footnotes

1 The first mentioning of a cosmetic use of *A. belladonna* seems to have been made by Ray et al. (1686-1704, p. 679) who wrote that the Venetians and other Italians call this plant belladonna because from its juice or with distilled water the women produce a juice they apply to their face and from red it turns pale, due to its cold power [sic]. Köhler (1887, p. 10) noted: The name ‘belladonna’ was first mentioned in the 16th century in Venice. The name was used to describe a red makeup produced from the red sap of the berries. As a botanical attribute belladonna was first mentioned by Matthioli in his commentaries from 1558 (Köhler, 1887, p. 10). In the edition from 1568 Matthioli writes that some call it ‘Solatro maggiore’ and others, such as the Venetians, ‘belladonna’ (p. 1131). It was, however, impossible for us to track down a historic source reporting the use of applying *A. belladonna* juice to the eyes. The fact that women do not show the same degree of attractions toward sexual arousal and increased pupil sizes as men could explain why *A. belladonna* juice was apparently not used by men subjects to increase pupil size (see Tombs and Silverman, 2004).

2 Being able to distinguish between what is right and wrong, Adam and Eve had acquired the moral concept to cope with the complex social and legal order of an agriculturalists’ society (e.g. Code of Hammurabi). While hunter-gatherer societies were not grounded on property and the accumulation of wealth was unknown, the transition toward agriculturalists’ societies saw the evolution of land tenure systems, ownership and heredity. In turn, for men the importance of determining their fatherhood increased. In the attempt to defend and secure family property, marriage and reproduction also received a strategic dimension and societies “began to adopt measures that would help assure paternity by developing new rules to govern female sexuality” (Stearns, 2009, p. 15–16). In parallel, religion evolved from animism via polytheism to monotheism (Theissen, 2007; Peoples et al., 2016). To a certain extent, however, animism, polytheism and monotheism can coexist as evidenced by the animism in Greek mythology (see Introduction) and the phenomenon of objectophilia or object sexuality (the emotional and sexual attraction toward inanimate objects; see e.g., Motschenbacher, 2014).

3 Majun al-tiryaq al-Kabir [Avicenna, 2014a, vol. 5, p. 2]: Camphor 0.5 mithqal, Ambergris 1 dirham, Musk 1 dirham, Conesse 2 dirham [bark of *Holarrhena pubescens* Wall. ex G.Don.], Eaglewood 2 mithqal [*Aquilaria* sp.], pepper seeds 2 dirham [*Piper* sp.], rocket seeds 2 dirham [*Eruca* sp. or *Diplotaxis* sp.], turnip seeds 2 dirham [*Brassica rapa* L.], Valerian 2.5 dirham [*Valeriana* sp.], dogwood 2.5 dirham [*Cornus* sp.], rock parsley 2.5 dirham [*Petroselinum crispum* (Mill.) Fuss], absinth 3 dirham [*Artemisia* sp.], cinnamon 3 dirham [*Cinnamomum* sp.], Nard 3 dirham [*Nardostachys jatamansi* (D.Don) DC.], Opium 3 dirham [*Papaver somniferum* L.], saffron 3 dirham [*Crocus sativus* L.], Sweet scented flag 3 dirham [*Acorus calamus* L.], Balm – leaves and seeds of cultivated, 4 dirham [*Melissa officinalis* L.], balsam seeds [?] 4 dirham, basil seeds 4 dirham [*Ocimum* sp], citron peels 4 dirham [*Citrus* sp.], doronic 4 dirham [*Doronicum* sp.], Genciana 4 dirham [*Genziana* sp.], Long Zedoary 4 dirham [*Curcuma* sp.], Myrrh 4 dirham [*Commiphora myrrha* (Nees) Engl.], Costus roots 5 mithqal [*Saussurea costus* (Falc.) Lipsch.]. It should be left for 6 month to mature and then used [1 dirham = ca. 3.2 g; 1 mithqal = ca. 4.25 g].
